# In Search of the Trauma Memory: A Meta-Analysis of Functional Neuroimaging Studies of Symptom Provocation in Posttraumatic Stress Disorder (PTSD)

**DOI:** 10.1371/journal.pone.0058150

**Published:** 2013-03-25

**Authors:** Gudrun Sartory, Jan Cwik, Helge Knuppertz, Benjamin Schürholt, Morena Lebens, Rüdiger J. Seitz, Ralf Schulze

**Affiliations:** 1 Clinical Psychology Unit, Department of Psychology, University of Wuppertal, Wuppertal, Germany; 2 Neurology Clinic, University of Düsseldorf, Düsseldorf, Germany; 3 Statistics Unit, Department of Psychology, University of Wuppertal, Wuppertal, Germany; Bellvitge Biomedical Research Institute-IDIBELL, Spain

## Abstract

Notwithstanding some discrepancy between results from neuroimaging studies of symptom provocation in posttraumatic stress disorder (PTSD), there is broad agreement as to the neural circuit underlying this disorder. It is thought to be characterized by an exaggerated amygdalar and decreased medial prefrontal activation to which the elevated anxiety state and concomitant inadequate emotional regulation are attributed. However, the proposed circuit falls short of accounting for the main symptom, unique among anxiety disorders to PTSD, namely, reexperiencing the precipitating event in the form of recurrent, distressing images and recollections. Owing to the technical demands, neuroimaging studies are usually carried out with small sample sizes. A meta-analysis of their findings is more likely to cast light on the involved cortical areas. Coordinate-based meta-analyses employing ES-SDM (Effect Size Signed Differential Mapping) were carried out on 19 studies with 274 PTSD patients. Thirteen of the studies included 145 trauma-exposed control participants. Comparisons between reactions to trauma-related stimuli and a control condition and group comparison of reactions to the trauma-related stimuli were submitted to meta-analysis. Compared to controls and the neutral condition, PTSD patients showed significant activation of the mid-line retrosplenial cortex and precuneus in response to trauma-related stimuli. These midline areas have been implicated in self-referential processing and salient autobiographical memory. PTSD patients also evidenced hyperactivation of the pregenual/anterior cingulate gyrus and bilateral amygdala to trauma-relevant, compared to neutral, stimuli. Patients showed significantly less activation than controls in sensory association areas such as the bilateral temporal gyri and extrastriate area which may indicate that the patients’ attention was diverted from the presented stimuli by being focused on the elicited trauma memory. Being involved in associative learning and priming, the retrosplenial cortex may have an important function in relation to trauma memory, in particular, the intrusive reexperiencing of the traumatic event.

## Introduction

Posttraumatic stress disorder (PTSD) is a severe anxiety disorder following a traumatic event. The diagnostic criteria are re-experiencing of the trauma, autonomic reactivity to and avoidance of trauma-related cues and elevated arousal [1]. The neuronal circuit proposed to underlie PTSD implicates hyperresponsivity of the amygdala, whose activity cannot be regulated by concomitantly hyporeactive medial prefrontal and anterior cingulate cortex (ACC), and a deficient hippocampal function preventing the re-assessment of the traumatic event [2]–[4]. The model inspired neuroimaging studies using SPECT, PET and fMRI to uncover the neuronal response to symptom provocation, i.e., the presentation of trauma reminders such as pictures, sounds or script-driven imagery. To data, however, results have been only partly convergent and there have been findings of additionally activated areas which did not form part of the proposed circuit [5]–[6].

Having been shown to be essential for fear conditioning [7], the amygdala was a frequent target of hypothesis-guided region-of-interest (ROI) analyses in neuroimaging studies of PTSD. In most studies, the amygdala showed hyperresponsivity during presentation of trauma narratives [8]–[9], to combat sounds [10]–[11], and combat pictures [12] in war veterans with PTSD. But there are also a number of reports of the amygdala showing no response to trauma-related cues (e.g. [13]–[14]) or even hyporesponsivity [15]. A hyperreactive amygdala is, in any case, not specific to PTSD but has also been found in other anxiety disorders [16], with generally aversive stimuli [17] and even during appetitive learning [18].

Medial prefrontal cortex/ACC is thought to have a regulatory function in emotional processing [19] which is impaired in PTSD. Consistent with this hypothesis, ACC was found to be less activated in PTSD patients than controls during symptom provocation in a number of studies [14], [20]–[24]. There were some exceptions, however [8], [25]. Compared to other anxiety disorders, hypoactivation in the dorsal and the rostral cingulate and ventromedial prefrontal cortex was specific to PTSD [16] but similar results were found in depression (e.g. [26]).

Results regarding hippocampal/parahippocampal involvement have been similarly inconsistent. Bremner et al. [21] found decreased hippocampal activation during script driven imagery in survivors of childhood sexual abuse and there are reports of impaired learning in PTSD patients [9], [27]. Conversely, other studies found evidence of increased hippocampal activation in PTSD (e.g. [28]) or could not replicate the finding of an impaired memory function [29].

Various factors have been suggested to account for the inconsistent results. There is considerable heterogeneity among PTSD patients regarding comorbid disorders. Nearly 50% of PTSD patients develop depression and almost as many suffer from alcohol/drug abuse and dependence [30]. The latter are particularly prevalent among long-term PTSD patients such as war veterans and victims of childhood abuse. Depression could account for hyporeactivity of the medial frontal/ACC area and alcohol/drug abuse for the abnormal functioning of the hippocampus.

It is noteworthy that the clinical features which most clearly distinguish PTSD from other anxiety disorders namely, reexperiencing and flashbacks, are not accounted for by the hypothesized neuronal circuitry. Reexperiencing has been proposed as the result of associative learning of the event with concomitant environmental stimuli which are subsequently able to evoke the trauma memory. The sudden occurrence of flashbacks has been explained by priming, an increased sensitization to features of trauma-related and associated stimuli [31]–[32]. These processes require activation of associative cortical areas which has been reported in a number of studies [12], [21], [22], [33] but, with a notable exception [4], have not been included in the hypothesized underlying neuronal circuit.

Owing to the considerable technical demands, only a small number of patients were included in most of the neuroimaging studies. By combining studies, meta-analyses may provide more reliable results. A number of meta-analyses of functional neuroimaging studies in PTSD have been carried out recently [16], [34]–[36]. Etkin and Wager [16] compared PTSD with other anxiety disorders and found amygdalar hyperactivation in all disorders but dorsal and rostral cingulate hypoactivation only in PTSD. Assuming that PTSD results in fundamental alteration of brain function, Patel et al. [34] and Simmons and Matthews [35] carried out meta-analyses across a variety of conditions and cognitive tasks. Patel et al. [34] confirmed the activation pattern reported by Etkin and Wager [16] and found additional hyperactivation in the hippocampus. Simmon and Matthews’ study [35] aimed at disentangling PTSD from mild traumatic brain injury. The authors found a potential overlap between the two disorders in findings on the middle frontal gyrus and also noted that some of the findings were task-specific. Finally, Haynes et al. [36] carried out separate meta-analyses of symptom provocation and cognitive task studies. Symptom provocation resulted in hyperactivity of mid- and dorsal anterior cingulate and hypoactivity of the medial frontal gyrus in PTSD patients.

The previous meta-analyses did not include all neuroimaging studies of symptom provocation. To date there are additional studies with considerably larger samples than were included so far. A further meta-analysis therefore appeared justified. Only symptom provocation studies have been included in the present meta-analyses, i.e. presentation of trauma related scripts or stimuli compared to a neutral condition. In addition, a further type of meta-analysis was employed which may be more appropriate to a combined analysis of widely varying samples.

Previous studies employed activation likelihood estimation (ALE) [37]–[38], the first method based on the regional likelihood of reported peak locations of significant activation clusters. A more recently developed coordinate-based method called signed differential mapping (SDM) [39] improved on the previous method by accounting for both hyper- and hypoactivation. A further development, Effect Size-SDM (ES-SDM) [40] combines peak coordinates and statistical parametric maps. This has improved both the overlap and sensitivity, while protecting against false negatives. ES-SDM was found to be superior to ALE in these respects [40].

In the present study, an ES-SDM meta-analysis was carried out comparing PTSD patients and trauma-exposed controls with respect to their pattern of neural activation to trauma-related stimuli. Further analyses compared reactions to trauma-related stimuli with the neutral condition within the patient and control group separately. We expected to confirm the hypothesized neuronal circuit and additionally, to find activation of associative cortical areas in patients but not in controls.

## Materials and Methods

### Study Selection

A literature search was conducted to identify fMRI, PET and SPECT studies of symptom provocation in traumatized individuals. The search for neuroimaging data was conducted on Medline, PubMed and Psychinfo in mid-January 2012. The search terms were: PTSD or ASD (acute stress disorder) + symptom provocation + PET (positron emission tomography), SPECT (single photon emission computerized tomography) or fMRI (functional magnetic resonance imaging). In addition, the reference lists of resulting articles were reviewed for relevant studies not identified by the initial database search. The search yielded 24 studies from which a subset was selected according to the following inclusion criteria: (1) PTSD or ASD diagnosis in the patient group, (2) symptom provocation, i.e., the use of trauma-related stimuli and a control condition.

The studies were additionally checked to ensure that the reported results were from independent samples. If samples were found to be overlapping, only data from the most recent report were included. Nineteen studies with a total of 274 patients met the inclusion criteria, 13 of which also presented data on a total of 145 trauma-exposed controls. A list of the studies with design characteristics is shown in table 1.

**Table 1:** Studies included in the meta-analyses with a description of the participants (men/women, index trauma, mean age (SD)) and method of symptom provocation.

First author	PTSD patients	Controls	Symptom provocation	Control condition	Method
[8] Rauch	2 m/6 w, mixed, 41.1 (3.4)	–	Personalized trauma script	Neutral script	PET
[20] Shin	7 m, vets., 45.4 (2.0)	7 m, vets., 50.0 (2.6)	Combat pictures	Neutral pictures	PET
[14] Shin	8 w, sex. ab., 37.1 (13.5)	8 w, sex. ab., 37.5 (8.3)	Personalized trauma script	Neutral script	PET
[78] Bremner	10 m, vets., 47.0 (3.0)	10 m, vets., 50.0 (3.0)	Combat pictures, sounds	Neutral pictures	PET
[21] Bremner	10 w, sex. ab., 35.0 (6.0)	12 w, sex. ab., 32.0 (8.)	Personalized trauma script	Neutral script	PET
[10] Liberzon	14 m, vets., 46.9 (.89)	11 m, vets., 51.2 (1.7)	Combat sounds	White noise	SPECT
[11] Pissiota	7 m, vets., 37.7 (28–52)	–	Combat sounds	Neutral tones	PET
[12] Hendler	10 m. vets., (22–62)	11 m, vets., (22–62)	Combat pictures	Civilian pictures	fMRI 1.5-T
[87] Driessen	6 w, mixed, 38.8 (6.2)	–	Trauma recall	Aversive recall	fMRI 1.5-T
[88] Lanius	11 mixed, 36.0 (12.0)	13 mixed, 34.0 (13.0)	Trauma recall	Neutral recall	fMRI 4-T
[9] Shin	7 m/10 w, vets., 51.6 (5.2)/51.8 (1.9)	9 m/10 w,vets., 54.9 (2.7)/51.6 (1.6)	Personalized trauma script	Neutral script	PET
[22] Yang	1 m/4 w, earth quake, 13.5	3 m/3 w, earth quake, 13.4	Trauma pictures	Neutral pictures	fMRI 1.5-T
[41] Lanius	10 w, mixed, dissociation 36.0 (12.0)	1 m/9 w, mixed 37.7 (11.1)	Personalized trauma script	Neutral script	fMRI 4-T
	2 m/9 w, mixed, flashbacks 35.2 (12.3)		and recall	and recall	
[15] Britton	16 m, vets., 53.8 (4.2)	15 m, vets., 56.5 (4.9)	Personalized trauma script	Neutral script	PET
[79] Hopper	7 m/20 w, mixed 35.9 (10.5)	–	Personalized trauma script	Neutral script	fMRI 4-T
[24] Hou	10 m, mining, 34.3 (4.5)	7 m, mining, 40.6 (5.3)	Trauma pictures	Neutral pictures	fMRI 1.5-T
[81] Lanius	4 m/11 w, mixed with MDD 34.6 (10.5)	3 m/13 w, 33.8 (12.1)	Personalized trauma script	Rest	fMRI 4-T
	4 m/4 w mixed without MDD 35.6 (9.9)				
[74] Moray	39 m, vets., 35.9 (9.4)		Combat pictures	Neutral pictures	fMRI 3-T
[82] Osuch	12 m/10 w MVA 32.5 (12.8)		Trauma script	Neutral script	PET

Note: vets. – war veterans; sex.ab. – sexual abuse; BDP – borderline personality disorder; MDD – major depressive disorder; MVA – motor vehicle accident.

Studies presenting threatening stimuli not directly related to the trauma, such as angry faces, or cognitive tasks were not included in the meta-analysis. Symptom provocation studies were included irrespective of whether stimuli were autobiographical or generic, e.g. combat noise in veterans. In case of multiple symptom provocation methods, preference was given to results from the presentation of pictures followed by imagery and trauma scripts. As to trauma pictures, only data from presentations above the perceptual threshold were included. Thus, in Hendler et al.’s study [12], only the response to pictures presented for 80 ms was included. Only one data set per study was included unless there were independent samples of patients, for example, PTSD patients with and without dissociation [41].

### Data Analysis

Three meta-analyses were performed comparing (1) PTSD patients with controls with regard to reactions to trauma-related stimuli, (2) reactions to the trauma-related with the neutral condition in PTSD and (3) controls, respectively. The meta-analyses were performed using ES-SDM (Effect Size Signed Differential Mapping) [40], [42]–[43] (http://www.sdmproject.com/software). The analysis method combines both peak coordinates and statistical parametric maps while also using standard effect size and variance-based meta-analytic calculations. First, reported peak coordinates were used to recreate a statistical map of the differences between groups/conditions for each study. MNI coordinates were converted into Talairach coordinates. Second, meta-analytic maps were obtained by voxel-wise calculation of the statistics of interest from the study maps, weighted by the squared root of the sample size of each study so that studies with large sample sizes contributed more. Additional control for heterogeneity between studies was provided by adopting a random-effect model. ES- SDM calculates both positive and negative differences between comparison conditions. A correction threshold of p<.005 and a cluster-size >10 voxel was applied to the results. A threshold of uncorrected p<.005 was reported to balance sensitivity and specificity optimally and to be an approximate equivalent to a corrected p<.05 in ES-DSM [40].

Peak coordinates were submitted to MRICroGL (http://www.cabiatl.com/mricrogl/) which provided templates to visualize the results.

## Results

**Figure 1:**
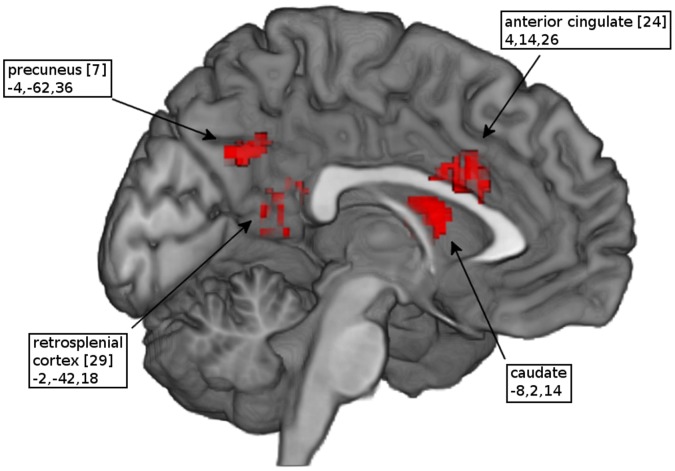
Activation map of patients contrasted with controls. Significant activations of PTSD patients compared to trauma-exposed controls in response to trauma-related stimuli. (Numbers in brackets indicate Brodmann areas and coordinates of the peak voxel are in Talairach space).

**Figure 2:**
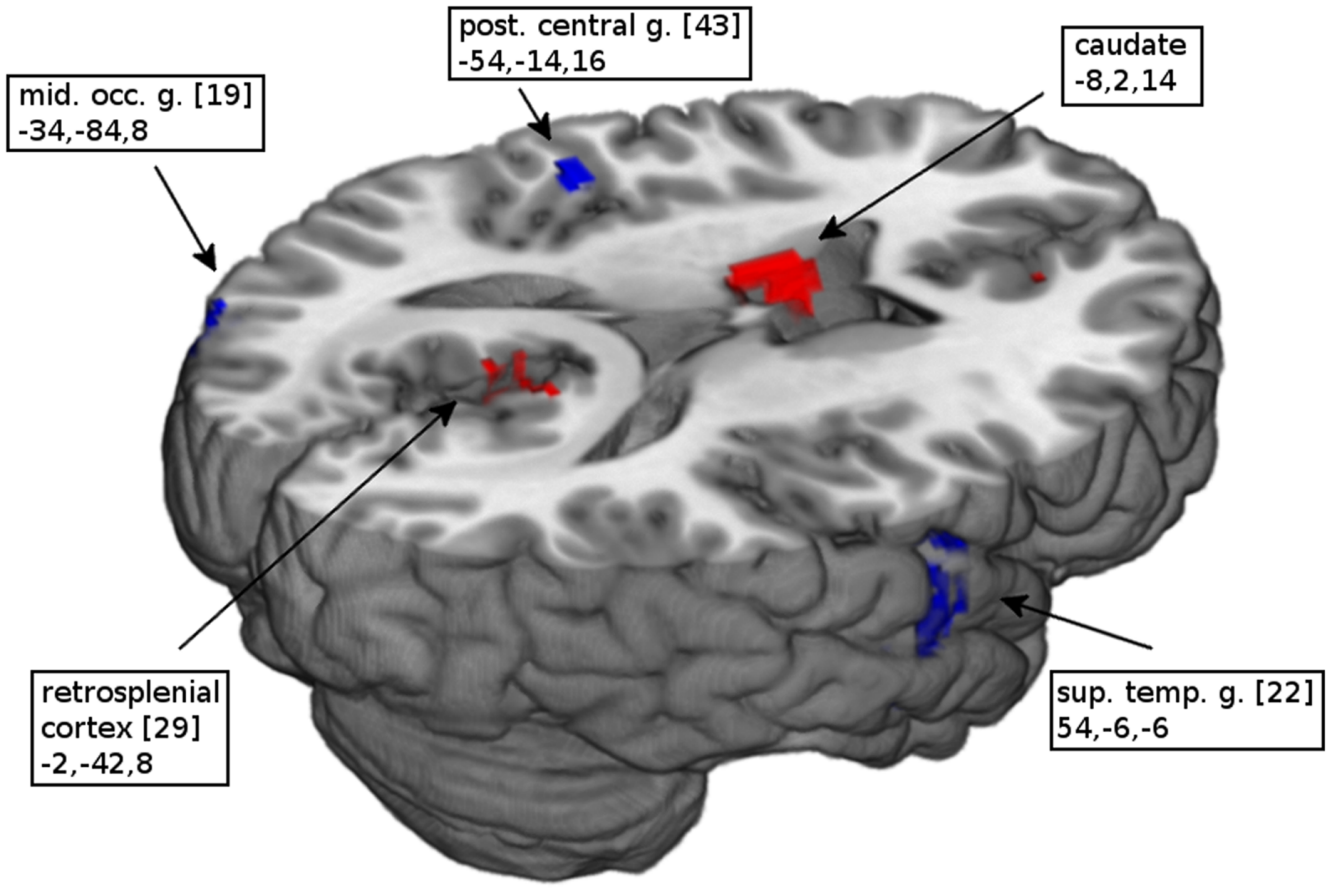
Activation map of controls contrasted with patients. Significantly increased (red) and decreased (blue) activations in PTSD patients compared to trauma-exposed controls in response to trauma-related stimuli. (Numbers in brackets indicate Brodmann areas and coordinates of the peak voxel are in Talairach space).

**Figure 3:**
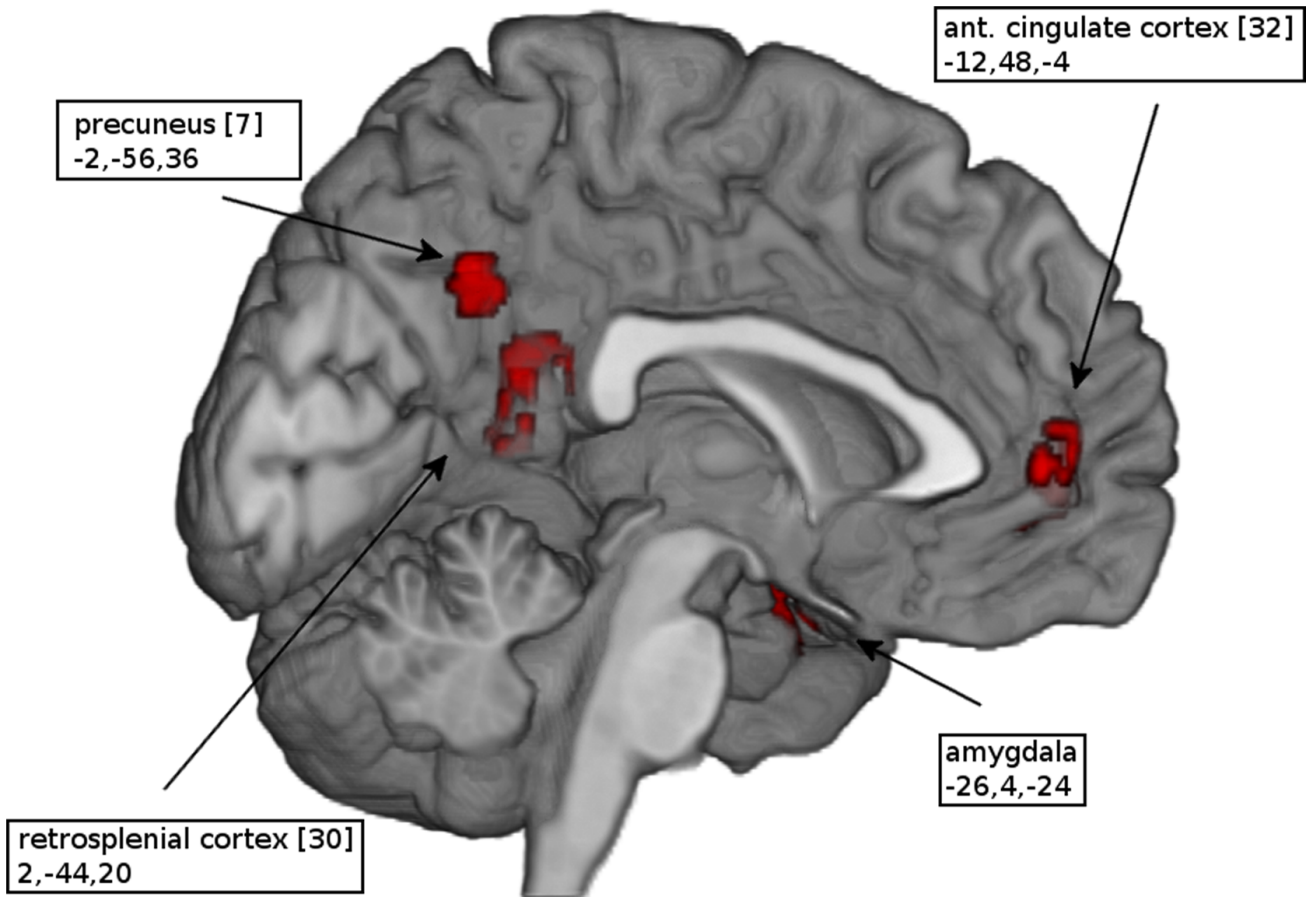
Activation map of patients. Significant activations of PTSD patients in response to trauma-related stimuli as compared to a neutral condition. (Numbers in brackets indicate Brodmann areas and coordinates of the peak voxel are in Talairach space).

**Figure 4:**
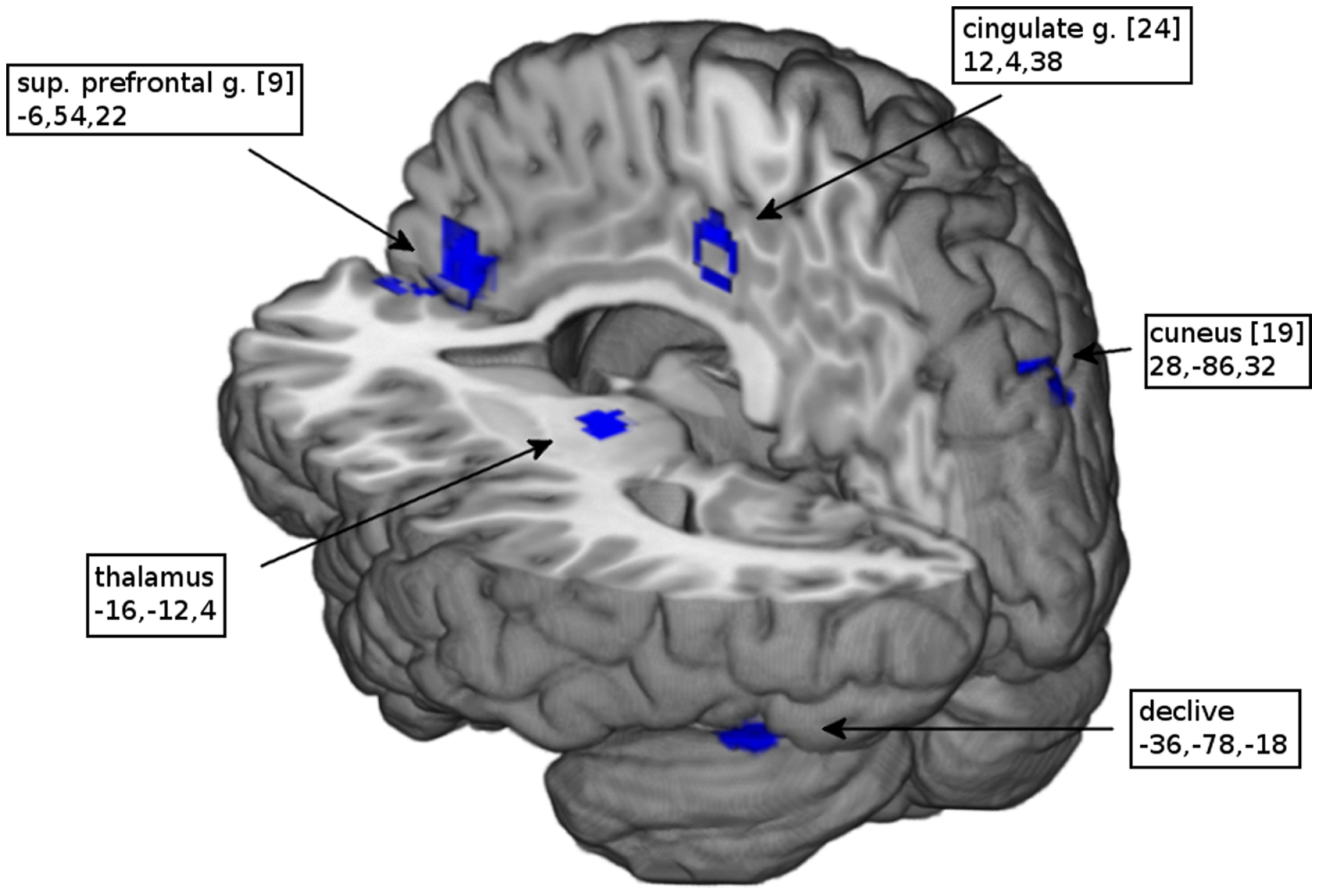
Activation map of controls. Significant activations of trauma-exposed controls in response to trauma-related stimuli as compared to a neutral condition. (Numbers in brackets indicate Brodmann areas and coordinates of the peak voxel are in Talairach space).

As shown in table 2, the comparison between PTSD patients and controls with respect to their response to trauma-related stimuli showed that patients exhibited greater activation than controls in the mid-line anterior cingulate cortex, retrosplenial cortex and precuneus (Fig. 1). Greater activation was also evident in the right middle frontal gyrus and superior parietal lobe as well as the left precentral and angular gyrus. In contrast, as shown in table 3, patients showed less activation than controls in superior and middle temporal gyrus, postcentral and mid-occipital gyrus (Fig. 2). When comparing trauma-related stimuli with the control condition (Table 4), patients exhibited significantly greater activation in the mid-line pregenual and retrosplenial cortex and precuneus (Fig. 3) as well as in the bilateral amygdala, mid-occipital and angular gyrus. In controls (Table 5), the most extensive activations were evident in midline superior prefrontal cortex together with left thalamus and bilateral dorsal cingulate gyrus, right cuneus and declive (Fig. 4). Comparing the two conditions, neither group showed significantly decreased activation.

**Table 2:** Comparison between PTSD patients and trauma-exposed controls in respect to their response to trauma-related stimuli (PTSD patients > controls).

Peak voxel					Additional local peaks				Cluster breakdown	
Description, BA	Talairach	*Z*	*P<*	Voxels	Description, BA	Talairach	*Z*	*P<*	Description, BA	Voxels>10
l.post.cingulate 29/	−2, −42, 18	2.297	0.001	100	l.post.cing. 29	−4, −46, 16	2.291	0.001	l.post.cing. 29	35
retrosplenial cortex					r.post.cing. 29	4, −42, 18	2.157	0.002	l.post.cing. 30	25
					l.post.cing. 29	−6, −46, 8	1.947	0.003	r.post.cing. 39	14
					r.post.cing. 29	6, −46, 16	1.889	0.003	l.post.cing. 23	11
					l.post.cing. 30	−6, −52, 6	1.771	0.004	r.post.cing. 30	10
l. precuneus 7	−4, −62, 36	1.869	0.003	37	l.precuneus 7	−2, −54, 38	1.859	0.003	l.precuneus 7	36
l.sup.temp.g. 39/g.angularis	−48, −56, 22	1.784	0.004	18						
l. caudate body	−8, 2, 14	2.409	0.001	291	l.ant.cing. 23	−2, 12, 24	2.284	0.001	l.caudate body	124
					r.cing.g. 24	4, 14, 26	2.158	0.002	l.ant.cing. 33	11
					l.cing.g. 24	−8, 12, 30	1.730	0.005	l.ant.cing. 24	15
									l.cing.g. 24	34
									r.cing.g. 24	28
									r.ant.cing. 24	11
									l.cing.g. 32	19
									r.cing.g. 32	11
r.medial frontal g. 9	6, 46, 20	1.926	0.003	23	r.medial f.g. 9	8, 50, 22	1.910	0.003	r.medial frontal g. 9	15
					r.superior f.g. 9	12, 48, 22	1.902	0.003		
					r.medial f.g. 9	10, 42, 16	1.734	0.005		
r. precuneus 7	32, −50, 48	2.091	0.002	45					r.sup.par.lob. 7	27
									r.precuneus 7	15
l.precent.g. 6/	−42, −10, 32	1.960	0.003	23	l.precent.g. 6	−38, −6, 36	1.817	0.004	l.precent.g. 6	23
frontal eye field					l.precent.g. 6	−42, −8, 36	1.782	0.004		

**Table 3:** Comparison between PTSD patients and trauma-exposed controls in respect to trauma-related stimuli (PTSD patients < controls).

Peak voxel					Additional local peaks				Cluster breakdown	
Description, BA	Talairach	*Z*	*P<*	Voxels	Description, BA	Talairach	*Z*	*P<*	Description, BA	Voxels>10
r.sup.temp.g. 22	54, −6, −6	−1.724	0.001	295	r.sup.tem.g. 22	56, −2, −6	−1.722	0.001	r.sup.tem.g. 22	127
					r.sup.tem.g. 22	56, 0, 4	−1.550	0.001	r.mid.tem.g. 21	66
					r.sup.tem.g.	60, 2, 0	−.441	0.002	r.sup.tem.g. 21	14
					r.mid.tem.g. 21	56, −10, −16	−1.390	0.002	r.sup.tem.g.	16
					r.inf.tem.g. 20	60, −12, −22	−1.354	0.002	r.precent.g. 6	11
					r.sup.tem.g. 22	50, −8, 0	−1.277	0.003	r.inf.tem.g. 20	20
					r.sup.tem.g. 22	52, −10, 4	−1.267	0.003	r.inf.tem.g. 21	23
					r.mid.tem.g. 21	62, −4, −12	−1.249	0.003	r.fusif.g. 20	10
					r.mid.tem.g. 21	64, −6, −6	−1.190	0.004		
					r.mid.tem.g. 21	58, −4, −16	−1.186	0.004		
					r.mid.tem.g. 21	58, −14, −10	−1.182	0.004		
					r.mid.tem.g. 21	62, −12, −12	−1.165	0.004		
					r.sup.tem.g. 22	48, −12, −2	−1.160	0.004		
l.mid.occ.g. 19	−34, −84, 8	−1.432	0.002	99	l.mid.occ.g. 19	−30, −90, 8	−1.187	0.004	l.mid.occ.g. 19	68
					l.mid.occ.g. 19	−30, −80, 18	−1.155	0.004	l.mid.occ.g. 18	28
					l.mid.occ.g. 19	−40, −82, 18	−1.150	0.004		
					l.mid.occ.g. 19	−42, −78, 12	−1.128	0.005		
					l.mid.occ.g. 19	−26, −88, 8	−1.119	0.005		
l.postcent.g. 43	−54, −14, 16	−1.402	0.002	38					l.postcent.g. 43	21
r.postcent.g. 4	58, −14, 30	−1.304	0.003	61	r.postcent.g. 2	58, −20, 30	−1.212	0.004	r.postcent.g. 3	20
									r.postcent.g. 4	24
									r.postcent.g. 2	11
l.mid.temp.g. 21	−62 −32 −12	−1.265	0.003	52	l.mid.tem.g. 21	−58 −28 −14	−1.265	0.003	l.mid.temp.g. 21	42
									l.inf.temp.g. 20	10

**Table 4:** PTSD patients: Comparison of reactions to trauma-related stimulation (symptom provocation) with a control condition.

Trauma > neutral condition peaks					Additional local peaks				Cluster breakdown	
Description, BA	Talairach	Z	P<	Voxels	Description, BA	Talairach	Z	P<	Description, BA	Voxels>10
r. posterior cingulate 30/	2, −44, 20	2.465	0.001	137	l. post.cingul. 23	−2, −46, 22	2.288	0.001	r. post.cingul. 30	22
retrosplenial cortex					r. post.cingul. 30	2, −48,16	2.128	0.001	l. post.cingul. 23	13
					l. post.cingul. 29	−4, −48, 6	1.505	0.003	l. post.cingul. 30	19
					l. culmen	−2, −48, 2	1.281	0.004	r. post.cingul. 23	18
									r. post.cingul. 29	17
									l. post.cingul. 29	29
l. anterior cingulate 32/	−12,48, −4	1.907	0.002	133	r. med.fron.g. 10	4,50,8	1.560	0.003	l. ant.cing. 32	28
pregenual cortex					l. ant. cingul. 32	−2,48,4	1.318	0.003	l. med. fr. g. 10	53
									r. m. front. g. 10	19
l. uncus 28/amygdala	−26, 4, −24	2.144	0.001	77	l. uncus amygd.	−26, −2, −22	1.289	0.004	l. uncus 28	33
									l. sup. temp.g 38	24
r. uncus 28/amygdala	32, 4, −22	1.814	0.002	81	r.parah.g.amyg.	24, −4, −18	1.662	0.002	r.parah.amyg.	47
					r.parah.g.amyg.	30, −2, −18	1.605	0.002	r. sup.temp.g 38	12
l. mid. occ. g. 18	−32, −84,2	1.865	0.002	49	l.mid.occ.g.19	−34, −82,6	1.628	0.002	l.mid.occ.g.18	30
					l.mid.occ.g.19	−38, −80,8	1.176	0.005	l.mid.occ.g.19	16
l. precuneus 7	−2, −56, 36	1.799	0.002	23					l. precuneus 7	22
l. mid. temp. g. 39/	−42, −60,20	1.278	0.004	10	l. mid.temp.g. 39	−46, −60,22	1.278	0.004		
g. angularis										

**Table 5:** Trauma-exposed controls: Comparison of reactions to trauma-related stimulation (symptom provocation) with a control condition.

Peak voxel					Additional local peaks				Cluster breakdown	
Description,BA	Talairach	*Z*	*P<*	Voxels	Description,BA	Talairach	*Z*	*P<*	Description,BA	Voxels>10
l. med. sup.fron. gyrus 9	−6,54, 22	3.088	0.001	282	l. sup.fron.g. 10	−6, 60, 22	2.898	0.001	l. sup. fron.g. 9	66
					l. sup. fron.g. 9	−8, 58, 26	2.869	0.001	l. med. fron.g. 10	79
					r. med. fron.g.10	4, 50, 12	2.155	0.002	l. sup. fron.g. 10	34
					l. med. fron.g. 10	−2, 54, 4	1.975	0.003	l. med. fron.g. 9	27
					l. med. fron.g. 10	−8, 58, 2	1.815	0.004	r. med. fron.g. 10	37
									r. med. fron.g. 9	38
l. thalamus	−16, −12, 4	2.528	0.001	115					l. thalam.	24
									l. thalam. VL	50
									l. m. glob. pall.	12
									l. lat. glob. pall.	11
l. declive	−36, − 78, −18	1.858	0.004	80					l. declive	78
l. cingulategyrus 24	−2, −16, 38	2.529	0.001	78	r.paracen.l.31	4, −20, 44	2.083	0.002	l. cingul. g. 24	27
					l.paracen.l.31	−2, −22, 44	2.081	0.002	l. paracen. l.	18
					r. paracen.l. 31	2, −16, 44	2.078	0.002	r. paracen. l.	15
					r. cingul.g. 24	4, −20, 36	1.931	0.003		
					r. cingul.g. 24	4, −16, 40	1.878	0.004		
					r. cingul.g. 31	6, −24, 40	1.829	0.004		
					l. cingul.g. 31	−4, −24, 40	1.813	0.004		
r. middle frontalgyrus 9	44,14,34	2.378	0.001	65					r. mid. fron.g. 9	52
									r. precent. g. 9	13
r. cuneus 19	28, −86, 32	2.418	0.001	59					r. cuneus 19	56
l. precentral g. 6	−44,0,30	2.044	0.002	28					l. precent. g. 6	19
r. cingulategyrus 24	12,4,38	1.971	0.003	26						

## Discussion

The coordinate-based meta-analysis of neural activation during trauma-related stimulation partly confirms and partly contradicts the current model of brain circuitry of PTSD. Furthermore, important additional areas were shown to be activated that hitherto have been neglected in the modeling of trauma symptoms. In patients, results from the between group and between condition analyses were widely overlapping.

Compared to controls and a control condition, PTSD patients showed significant activation of the mid-line retrosplenial cortex, precuneus, anterior cingulate gyrus as well as the left angular gyrus. Little attention has so far been paid to the role of activation of medial posterior aspects namely, retrosplenial cortex and precuneus in PTSD. The reevaluation of research into mid-line areas has recently produced new insights that are likely to be of relevance for our understanding of - among anxiety disorders – the unique and most salient of all PTSD symptoms namely, reexperiencing.

Activation of cortical midline structures during resting, i.e., the absence of stimulus-driven processing, has been considered to be indicative of the baseline of brain functioning [44] or a default mode network [45]–[46]. The use of self-related tasks during neuroimaging, e.g. when participants evaluated whether statements could be attributed to themselves [47]–[49] has, however, led to the suggestion that these areas subserve self-referential processing. Surveying the experimental evidence led Northoff and Bermpohl [50] to hypothesize a system whereby anterior midline areas are involved in the representation, evaluation and monitoring and posterior areas in the integration of self-referential stimuli and autobiographical memory. These processes appear to be independent of sensory modality and domain and were reported to be activated by tasks such as viewing a video of a previously experienced bank robbery [33] or hearing a familiar as compared to an unfamiliar voice [51]. Furthermore, recollecting familiar faces or events involving the self could be shown to result in activation of the retrosplenial cortex [52]–[54]. Meta-analyses of neuroimaging findings of autobiographical memory [55]–[56] confirmed the implication of this area. Further corroboration results from cognitive impairment of episodic memory and autobiographical amnesia which have been reported to be the result of damage to the retrosplenial cortex [57]–[58].

Activation of the retrosplenial cortex has also been shown to be essential for successful associative learning, i.e., forming of associations between multiple sensory stimuli in rodents [59] and, similarly in humans, for learning contextual associations and priming [60]–[62]. These processes are likely to be required in the formation of autobiographical memory and self-referential processing. Contextual associations and priming are considered to bring about reexperiencing of the traumatic event in PTSD [31]–[32] and could, therefore, also account for the activation of the retrosplenial cortex during symptom provocation procedures.

It is noteworthy that control participants failed to show significant activation of the retrosplenial cortex although they, too, were confronted with material of self-relevance. Two reasons could account for this group difference. PTSD patients may have shown greater activation because the trauma memory was more recent for them owing to their reexperiencing the event. The group difference is, however, more likely to be due to the trauma-related material being more salient for patients than controls. As a rule, the trauma-related material evoked more fear in patients than controls (e.g. [15]) and can therefore be considered to be more salient for the former. Reviewing the literature of retrosplenial cortex and emotion, Maddock [63] found that the former was more strongly activated by salient stimuli from autobiographical memory.

The present meta-analysis also revealed activation of the precuneus, the medial extent of area 7, viz. the anterior portion, in PTSD patients during symptom provocation and in comparison with control participants. Activation of the precuneus was found to be correlated with that of area 31 of the retrosplenial cortex [64] and reported to have a central role in a wide spectrum of highly integrated tasks. Reviewing the literature, Cavanna and Trimble [65] reported involvement in visual-spatial imagery, episodic memory retrieval and self-processing operations such as first-person perspective taking. The authors pointed out that the neuroimaging data suggest a dissociation of function within the precuneus with the anterior region being involved in self-referential processing and the posterior region subserving episodic memory retrieval. Comparing the BOLD response to pictures that were either self-referential or familiar, Sajonz et al. [66] confirmed that the former elicited activation in anterior and the latter in posterior portions of the precuneus. The present result of activation of the anterior precuneus would therefore suggest self-referential retrieval of trauma-related memories during symptom provocation in patients. Together with the retrosplenial cortex this area could play an essential role in the intrusive and distressing reexperiencing of the traumatic event in PTSD.

The left anterior cingulate gyrus is also considered to be part of the circuit subserving self-referential processing [65], [67], in particular, with regard to the judgment whether stimuli were self-referential [68]–[70]. This area has therefore been accorded a monitoring role [50]. Summarizing, findings of the activated midline structures to trauma-related material can be considered to be indicative of the adjudgment and retrieval of events with high self-relevance in PTSD patients.

The left gyrus angularis (area 39) was significantly activated during trauma-related stimulation in patients and significantly more so than in controls. The temporo-parietal junction is considered a high level centre integrating multisensory, sensorimotor and cognitive function [71] and has been accorded the role of a prominent node in the default/autobiographical memory network [72]–[73]. Morey et al. [74] reported a positive correlation between activation of this area and a global symptom severity score which leaves the question open as to the relationship to particular symptoms. It is conceivable that activation of the gyrus angularis induces dissociation. Stimulation of this area has been shown to induce out-of-the-body experiences [75] which are sometimes reported by PTSD patients as occurring during the most intensely stressful time of the traumatic event. The experience is usually accompanied by dissociation, mental and emotional disengagement.

Patients exhibited greater activation than controls in the left caudate body and dorsal anterior cingulate cortex (ACC). The latter has been found to be part of a network for salience processing [76] and is thought to form an essential node within the salience network [72]. The caudate nucleus is part of the system involved in motor function. Together with the activated frontal eye field (6) and the dorsal ACC, it is likely to form a circuit which gives rise to eye movement which, in turn, is part of the orienting reaction elicited by the fear evoking stimuli in patients.

The exaggerated amygdalar response has been found by the majority of neuroimaging studies of PTSD but also of other anxiety disorders [16]. Including only whole brain analyses, a recent meta-analysis of neural correlates of basic emotions found fear to be consistently activating both amygdalae and insula whereas responsivity of the latter was also involved in other emotions such as anger and disgust [77]. Unlike in these previous reports, no increased activation of insula was found among the present results. Both the previous meta-analyses included studies which employed a variety of emotion-related processes and cognitive tasks and are not, therefore, directly comparable with the narrow focus on symptom provocation of the present meta-analysis.

PTSD patients showed less activation than control participants with regard to lateral and dorsal sensory association areas namely, the left mid and right superior temporal gyrus, the left mid occipital gyrus and the bi-lateral postcentral gyrus. In two thirds of the studies stimuli used for symptom provocation consisted of personalized scripts of the traumatic event and in the remaining ones of pictures and sounds such as combat noise. Apart from being autobiographically relevant, trauma-related stimuli were generally unpleasant and arousing. Activation of the sensory association areas in controls suggests increased attention and processing of the presented material. It is conceivable that the self-referential processing in patients, unlike in controls, comes at a cost and has an inhibiting effect on the capacity to process concomitant environmental stimuli.

The present results failed to support the hypothesized inhibition of anterior cingulate cortex (ACC) activation. The area is thought to play an important role in emotional regulation and failure to do so in PTSD. The effect does not appear to be due to stimulus modality. Some studies reporting reduced activation presented pictures [22], [24], [79] and others personalized scripts [9], [15], [79]. But as many studies reported increased ACC activation with pictures [41], [74], [80] as with personalized scripts [14], [41], [81]–[82]. As PTSD is a heterogeneous disorder, ACC activation could be related to particular symptoms. Morey et al. [74] found increased ACC activation to be positively correlated with PTSD severity while Lanius et al. [41] reported increased ACC activation in PTSD patients suffering from flash-backs but not in patients with dissociation. In contrast, Hopper et al. [79] found decreased ACC activation to be related to reexperiencing. Decreased ACC activation may not be specific to anxiety disorders as it has also been found in depression [26], a frequent comorbid disorder of PTSD.

Both patients and controls showed significant activation of the medial superior prefrontal cortex (BA 9), patients more so than controls and the latter in response to trauma-related compared to neutral stimuli. It has been suggested that this area is associated with theory of mind, the ability to attribute mental states to others, and empathy, the ability to infer emotional experiences [83]–[86]. As is known from clinical experience, traumatic events frequently consist of witnessing injury or death of close friends, comrades or relatives. It could be speculated that the evocation of such trauma memories also elicits an empathetic response in the trauma survivors.

Among the limitations of this meta-analysis is the relatively small sample size of most of the studies included and their heterogeneity of the symptom provocation methods. Furthermore, additional analyses using control volunteers who had not undergone the traumatic event could have been informative in differentiating reactions to stimulus salience from autobiographical memory. However, as yet there are not enough studies involving unconcerned controls. Finally, heterogeneity among studies resulted from some of them reporting whole-brain analysis and others region-of-interest analysis. We hoped to compensate at least partly for the heterogeneity by using a random-effects model.

### Conclusions

Activation of the midline retrosplenial cortex and precuneus in response to symptom provocation suggests enhanced self-referential processing and evocation of salient autobiographical memory in PTSD patients. This appears to come at the cost of attending to the presented stimuli, scripts, pictures, noises, as evinced by the greater activation of auditory and visual association areas in trauma-exposed controls. The results suggest that the retrosplenial cortex has an important role in establishing and maintaining the trauma memory. Furthermore, its implication in associative learning and priming makes the retrosplenial cortex a likely candidate for giving rise to reexperiencing and intrusive memories of the precipitating traumatic event.
